# Molecular epidemiology of group A human rotaviruses in North West region, Cameroon

**Published:** 2012-08-15

**Authors:** Florence Azie Mbuh, George Enyimah Armah, Sunday Aremu Omilabu, Aliyu Ahmadu Ahmad, Jarlath Udoudo Umoh

**Affiliations:** 1 Ahmadu Bello University Zaria, Nigeria; 2 Noguchi Memorial Institute for Medical Research, University of Ghana, Legon, Ghana; 3 University of Lagos, Lagos, Nigeria

**Keywords:** Rotavirus, diarrhea, molecular epidemiology, electropherotypes

## Abstract

**Background:**

Rotavirus (RV) is the most common cause of severe diarrhea in children <5 years of age worldwide accounting for 527,000 deaths annually. Over 80% of these deaths occur in South Asia and sub-Saharan Africa. RV vaccines have significantly reduced RV-associated morbidity and mortalities in several countries like the United States and Mexico while vaccine trials have proved efficacious in Ghana and other developing countries. However, there is paucity of data on RV infection in Cameroon where diarrhea is a major childhood disease.

**Methods:**

A total of 534 stool specimens collected between January 2003 and December 2004 from children with acute gastroenteritis in five health districts in the NWR of Cameroon were screened for group A human rotavirus antigen by ELISA and their electropherotypes determined by Polyacrylamide gel electrophoresis.

**Results:**

RV was detected in 153 (28.7%) diarrheic specimens with infection occurring throughout the year, being more common in children under two years of age (P < 0.01) with the highest incidence in the 7-9 months age group (P <0.05). Sub clinical infections (9%) occurred mostly in children aged 0 - 6 months old (P<0.01). Source of drinking water was not associated with RV infection. Eleven electropherotype patterns were detected with predominance of long electropherotypes (92.8%) and mixed electropherotypes were seen only in hospitalized children. Some isolates showed overlapping or merged genome segments 7 and 8 or 9 and presenting with 10 segments of the RV genome.

**Conclusion:**

RV is a significant cause of pediatric diarrhea in the NWR affecting mostly children under 2 years of age. Continuous RV surveillance and nationwide surveys are recommended to improve the health of young children in Cameroon. More research is needed to fully characterize the isolated RV strains.

## Background

Rotavirus (RV) infection is a major cause of infantile diarrhea worldwide resulting in about 527,000deaths yearly among children under 5 years of age [1]. In tropical countries, the infection occurs throughout the year with increased prevalence during the dry season. Rotaviruses are transmitted by fecal-oral-route. Following an incubation period of 1 to 3 days the disease is characterized by acute onset of profuse watery diarrhea, fever, vomiting, irritability and lethargy. Diarrhea and vomiting are the most prominent symptoms, often leading to dehydration, shock and death if not treated [2]. Estimates show that about 1205 children die daily from rotavirus infection with over 80% of cases occurring in the poorest nations [3].

RV particle consists of an outer capsid, mid capsid and a core, which surrounds 11 segments of double stranded RNA (dsRNA) genome that codes for structural and non-structural viral proteins [4]. The intermediate capsid protein, VP6, is the most abundant protein of the virus and is important in the classification of RVs into seven serogroups (A-G). The VP6 protein is also the target of RV detection by enzyme immunoassay [5]. Differences in the molecular weight of the 11 segments of RV genome reflect in their separation on polyacrylamide gels. The 11 cognate segments typically migrate in groups of 4, 2, 3, 2 and strains from the same environment may show variations in their electropherotypes [6]. With respect to differences in the migration of genome segments 10 and 11 RVs are broadly grouped into long and short electropherotypes [7].

RV vaccines currently in use in developed nations have been shown to be cost effective for the prevention of severe RV disease in children in developing countries [8]. Efficacy studies showed a reduction in RV-associated diarrhea morbidity and mortalities in U.S, Mexico [9, 10] and some African countries [11-13]. However, in order to evaluate vaccine efficacy the epidemiology of rotavirus is needed before and after the introduction of new vaccines [2]. The only report on RV in Cameroon conducted in the West and South West Regions established a 21.9 % prevalence of RV infection among children under 5 years and a predominance of long electropherotypes [14]. This study was undertaken to provide baseline data on the epidemiology of RV infection in the North West Region (NWR) of Cameroon and to upgrade the limited information on RV diarrhea in the country.

## Methods

### Study area and population

The study was conducted in the NWR of Cameroon located between latitude 5° 20’ and 7°10’ N and longitudes 9° 4’ and 11° 15’ E with altitude range of 300m to 3000m above sea level. Temperature varies within the year between 10°C and 28°C and mean annual rainfall is 2000mm, while relative humidity ranges from 49% to 87% [15]. There are two alternative seasons: the rainy season begins from mid-March to October and the dry season covers the rest of the year. The region has a population density of 69 inhabitants / km^2^ with a total of 230,328 children under 5 years old (NID statistic 2003). There are no major industries and most inhabitants are mainly subsistent farmers producing mostly cereals, tubers, bananas and fruit trees. Apart from malaria, diarrhea is the most common cause of hospitalization among children under 5 years old and is also among the top 5 causes of death in this age group (Department of Statistics, North West Regional Delegation of Health, 2003, 2009).

### Samples collection and handling

Stool samples were collected from 534 children < 5 years with acute diarrhea who sought medical attention at clinics and hospitals in the NWR between January and December 2004. In addition, 134 non- diarrheic stool samples were collected as control. An interviewer-assisted structured questionnaire was administered to every parent or guardian submitting a stool specimen to obtain basic demographic and clinical information of the children. Stool samples were collected from the Regional hospital in Bamenda, District hospitals in Bafut, Bali, Batibo, Ndop and Santa, and from the Catholic Health Centre, Won and Presbyterian Health Centre, Nsem. Stool samples were stored at -20° C and transported on ice to the Virology Research Unit, College of Medicine of the University of Lagos, Nigeria for group A RV screening and to the West African Regional Rotavirus Laboratory, University of Ghana, for further analysis.

### Detection of RV by ELISA

RV antigen was detected by enzyme immunoassay on 10% fecal suspension in phosphate buffered saline using commercial IDEIA-Rotavirus kit (DAKO, Sweden) following the manufacturer's instructions. Briefly, a 10% suspension of each fecal material was made in phosphate buffered saline (PBS) pH 7.2 and 100µl of each suspension was dispensed into each of 96 wells of the microliter plate coated with anti-human rotavirus coating antibody. The first four wells contained a blank, 2 negative controls and a positive control respectively. Test he tests were read visually

### Polyacrylamide gel electrophoresis (PAGE)

RV genome was extracted by the Bender Buffer (BF) method outlined in the Regional Rotavirus laboratory manual. Briefly, 200 µl of 10% stool suspension in PBS were mixed with 200µl of BF (0.58g Sodium Chloride, 6.8g sucrose, 10 ml 1 M Tris-HCl pH 8.0, 50ml 0.1M EDTA, 2.5ml 20% SDS) incubated at 65° C for 30 min, and 8M potassium acetate was added. RNA was precipitated in alcohol, electrophoresed overnight and visualized by silver nitrate staining [16]. Specimens with incomplete bands were considered as negative for RV genome.

### Statistical analysis

Odds ratios were calculated for RV infection in children by age and other variables using the 2003 version of EPI-Info software and differences with p-values > 0.05 at 95% confidence intervals were not considered significant.

## Results

RV antigen was detected in 28.7% of diarrheic stool specimens and in 9.0% of control samples, (Table 1). Children 7-9 months old had the highest age specific rate (40.0%), (OR= 1.91; 95% CI: 1.19 < OR < 3.6; P< OR = 6.83; P < 0.001). Infection was more common in males but the difference was not statistically significant.

**Table 1:** Age distribution of rotavirus infection in children < 5 years of age, North West Region, Cameroon

Age Group (months)	Diarrheic Children			Non Diarrheic Children		
	Number tested	Number positive	% positive	Number tested	Number positive	**%**
0 – 3	18	6	33.3	8	3	37.5
4 – 6	48	12	25	14	3	21.4
7 – 9	105	48	40	17	0	0
10 – 12	92	33	35.9	11	1	9.1
13 – 18	110	31	28.2	24	0	0
19 – 24	60	17	28.3	13	1	7.7
25 – 60	101	12	11.9	47	4	8.5
Total	534	153	28.7	134	12	9

Younger children (0-3 months) had the highest asymptomatic RV shedding (37.5%) compared with other age groups (OR= 7.8; 95% CI: 1.23 < OR< 8.6; P<0.01). Generally ages 0-6 months were significantly associated with asymptomatic RV infection (OR= 6.63; 95 % C1: 1.63 < OR < 27.26; P<0.01). Subclinical infections were not detected in the 7-9 and 13-18 months age group.

Table 2 shows some characteristics features of RV infected children. Majority of patients presented with vomiting, watery stools and dehydration. RV infection was significantly associated with diarrhea hospitalization compared with diarrheic children treated at the outpatient department (OR = 3.05, 95% CI: 1.66 < OR < 4.67; P < 0.001). Source of potable water had no effect on the prevalence of RV infection but 73% of cases occurred in children whose mothers had little or no education.

**Table 2:** Clinical characteristics of diarrheic children < 5 years old, North West Region, Cameroon

Characteristic	No. Tested	Rotavirus positive	Percent
**General Condition**			
Normal	18	8	5.2
Ill	403	103	67.3
Very ill	112	42	27.5
**Mental status**		-	-
Normal	334	96	62.7
Irritable	59	15	9.8
Drowsy/lethargic	140	42	27.5
**Fever**	389	121	79.1
**Vomiting**	389	129	84.3
**Mean freq. Of vomiting** *	2.9	3.5	-
**Mean freq. Of diarrheic stool** *	5.7	6.6	-
**Dehydration**	213	100	65.4
**Hospitalized**	277	107	69.9
**Stool consistency**			
Watery	218	96	62.7
Loose	295	55	35.9
Normal	21	2	1.3
**Drinking water source**			
Spring	271	81	54.5
Pipe borne	263	70	45.5
**Mothers education**			
Up to primary school	387	112	73.2
Secondary and above	147	41	73.2

* On worst day of symptom

### Monthly distribution

**Figure 1:**
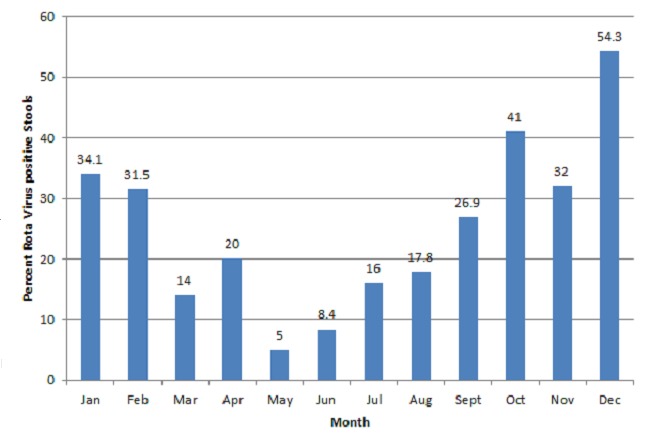
Monthly distribution of Rotavirus Diarrhea in the North West Region

RV infections occurred throughout the year with most cases occurring in the months of October through February (Figure 1). Peak and least month-specific prevalencewas seen in December(54.3) and May (5.0%) respectively.

### Polyacrylamide gel electrophoresis (PAGE)

**Figure 2:**
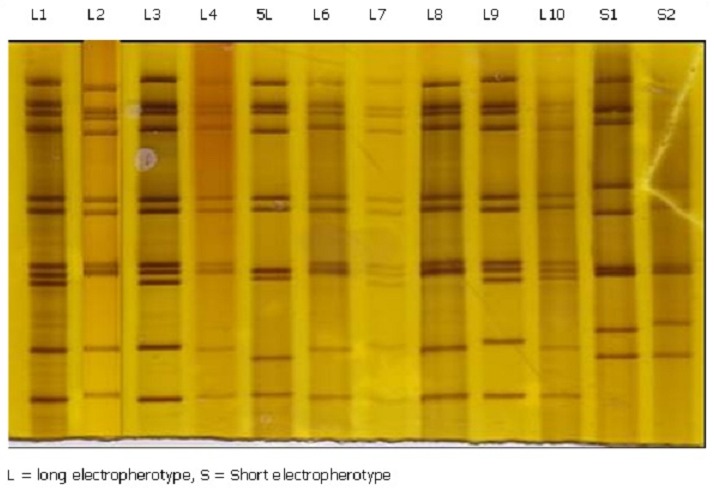
Rotavirus electropherotype patterns detected in children with diarrhea

RV genome was detected in 82/128 (64.1%) of the RV-ELISA positive specimens tested. Seven specimens showed incomplete segments and were regarded as negative. Long and short electropherotypes were detected in 76 (92.8%) and 5 (6.1%) isolates respectively. Mixed infections were detected in 10 (12.2%) stool specimens comprising nine with mixed long electropherotypes and one with both long and short patterns. All mixed infections were seen in hospitalized children. Twelve different migration patterns comprising 9 long (L) and 2 short (S) electropherotypes were identified with strains showing different migration patterns of the genome segments in both long and short electropherotypes (Figure 2). Patterns in L1 represented the most common long eletrophenotypes (36.6%), followed by L5 (16.6%), L3 (9.1%), L7 and L9 (6.1% each). Differences in migration patterns of strains with long electropherotypes were seen in cogent genome segments 7, 9, and 10. The L5 strains had the faster migrating genome segment 10. Strains represented by L2, L4, L5, L6, S1 and S2 showed patterns with either merged segments 7 and 8 or segments 8 and 9 and appearing to have only 10 genome segments. For the short electropherotypes, strains represented by S2 had larger genome segments 6 and 10 evident by slower migration on PAGE.

All short electropherotypes occurred in children aged 7-18 months. The mixed electropherotypes were seen in children aged between 6-21 months from Bamenda 5 (50%), Bafut 4 (40%), and Bali, 1 (10%) health districts. The specimen with both genome long and short profiles was from a 16 months old male child from Bafut health District. Children with mixed infected had a longer duration of hospital stay, ranged from 4-17 days (mean = 8 days). Generally, mixed infections were more common in the 13 - 18 months age group (Table 3). Of the 82 specimens with demonstrable rotavirus genome, 39 (47.6%) were from homes using spring while 43 (52.4%) used pipe borne water. Fifty per cent of children with mixed infections were from homes using either spring or pipe born water for domestic purposes.

**Table 3:** Age and sex distribution of rotavirus electropherotypes from diarrheic children, North West Region, Cameroon

Age (months)	Male	Female	Total	Percent
**0 – 3**	4	0	4	4.9
**4 – 6**	5	2	7	8.5
**7 – 9**	10	16^b^	26	31.7
**10 – 12**	14^a^	6	20	24.4
**13 –18**	9^m^	7^c^	16	19.5
**19 – 24**	3	4	7	8.5
**25 – 60**	0	2	2	2.4
**Total**	44	38	82	100

* The five short and one mixed electropherotypes were distributed between ages 7 – 18 months as follows:

a a = 1 short

b b = 2 short

c c = 2 short

m m = 1 short + 1 long electropherotypes in the respective age groups. The rest of the electropherotypes were long.

## Discussion

The prevalence of RV diarrhea was consistent with the 15-40% reported among children 5]. Asymptomatic infection was higher than the 2-4% prevalence generally seen in Africa, though higher rates (35.5%) have been reported in Nigeria [17] indicating that the prevalence of both sub-clinical and symptomatic RV infections varies with geographical location. High prevalence of sub clinical infection shows the existence of a large reservoir of RV that could become potential sources for transmission to susceptible children. This is facilitated by large numbers of RV virus shed in stools and the virus can remain viable for hours on human hands and for days on fomites [18], possibly accounting for the high rate of spread among hospitalized children and those in day care centers.

Peak RV infections usually occur between 7-12 months [19]. Absence of asymptomatic infection in the 7-9 month age group may be due to their greatest susceptibility to clinical infection. Children gradually acquire immunity to the virus from repeated infections [3] which explains the fewer infections in older children. Most RV infections in the tropics usually occur in the dry and cooler months [20] which could be due to increased outdoor activities and exposure of children. Fever, vomiting and watery diarrhea are common clinical features in children with rotavirus diarrhea often leading to dehydration that can be fatal if not treated thus contributing to the high rate of mortality associated with rotavirus diarrhea in developing countries [3] where there are limited facilities for rehydration therapy.

The dominance of long electropherotype was consistent with findings in the sub region [14, 21] and elsewhere [22]. However, mixed infection with both long and short electropherotypes was observed in Cameroon for the first time. The co-circulation of different RV electropherotypes in one community is a common feature in most epidemiological studies with predominance of a single strain showing minor variations in genomic RNA profile [23]. Mixed infections could have resulted from a superimposed hospital acquired infection or community cases that produced more sever disease requiring inpatient care. The high diversity of RV genome electropherotypes is of epidemiological importance and could lead to evolution of more virulent strains. Some strains presented with 10 genome segments in groups of 4, 2, 2, 2, instead of the typical 11 segment rotavirus genome that migrate in groups of 4,2,2,2 on PAGE according to segment sizes [6]. It was not clear whether the apparent merging of cogent genome segments 7 and 8 or segments 8 and 9 were as a result of massive mutations in the genome segments thus affecting their migration or due to a complete deletion of a segment. These strains were not considered to be reoviruses because they migrate in bands of three large, three medium and four small segments [24, 25]. Differences in cultural, environmental conditions and time may have accounted for the low frequency of short electropherotypes compared with 25% reported in neighbouring West and South West regions [14]. RV strain diversity may results from reassortment and antigenic drift [26] where the former occur in an individual simultaneously infected with more than one strain of the virus and continuous subclinical infections could facilitate reassortment is further facilitated by in order children [27].

## Conclusion

Our study established a 28.7% prevalence of rotavirus diarrhea and 9.0% asymptomatic infections among children in the NWR. Improving female education in Cameroon could help in reducing RV associated morbidity among infected children because 73% of cases were those whose mothers had little or no education. Serotyping, genotyping and phylogenetic studies are required to further characterize the RV stains and those showing abnormal genome electropherotypes. A large pool of genomic variations calls for continuous surveillance of RV infection in NWR and other parts of the country and a need for a government sponsored nation-wide rotavirus research in Cameroon. Government should also consider including RV vaccine in the Expanded Program on Immunization (EPI).A manuscript for VP7 and VP4 genotype characterization of the isolated RV strains is in preparation.
